# The Zn-finger domain of MdmX suppresses cancer progression by promoting genome stability in p53-mutant cells

**DOI:** 10.1038/oncsis.2016.62

**Published:** 2016-10-03

**Authors:** Z Matijasevic, A Krzywicka-Racka, G Sluder, J Gallant, S N Jones

**Affiliations:** 1 Department of Cell and Developmental Biology, University of Massachusetts Medical School, Worcester, MA, USA

## Abstract

The *MDMX* (*MDM4*) oncogene is amplified or overexpressed in a significant percentage of human tumors. MDMX is thought to function as an oncoprotein by binding p53 tumor suppressor protein to inhibit p53-mediated transcription, and by complexing with MDM2 oncoprotein to promote MDM2-mediated degradation of p53. However, down-regulation or loss of functional MDMX has also been observed in a variety of human tumors that are mutated for p53, often correlating with more aggressive cancers and a worse patient prognosis. We have previously reported that endogenous levels of MdmX can suppress proliferation and promote pseudo-bipolar mitosis in primary and tumor cells derived from p53-deficient mice, and that MdmX-p53 double deficient mice succumb to spontaneously formed tumors more rapidly than p53-deficient mice. These results suggest that the MdmX oncoprotein may act as a tumor-suppressor in cancers with compromised p53 function. By using orthotopic transplantation and lung colonization assays in mice we now establish a p53-independent anti-oncogenic role for MdmX in tumor progression. We also demonstrate that the roles of MdmX in genome stability and in proliferation are two distinct functions encoded by the separate MdmX protein domains. The central Zn-finger domain suppresses multipolar mitosis and chromosome loss, whereas the carboxy-terminal RING domain suppresses proliferation of p53-deficient cells. Furthermore, we determine that it is the maintenance of genome stability that underlies MdmX role in suppression of tumorigenesis in hyperploid p53 mutant tumors. Our results offer a rationale for the increased metastatic potential of p53 mutant human cancers with aberrant MdmX function and provide a caveat for the application of anti-MdmX treatment of tumors with compromised p53 activity.

## Introduction

The p53 transcription factor regulates expression of wide variety of genes involved in cellular response to stress, and mutation of p53 gene is the most common genetic lesion in human cancers. The level of p53 activity in a cell is tightly regulated by the MDM2 and MDMX (MDM4) oncoproteins. These MDM homologs bind to the amino-terminal p53 transactivation domain and inhibit p53 regulation of heterologous gene expression. MDM2 and MDMX heterodimerize to facilitate MDM2-mediated ubiquitination and degradation of p53.^1, 2, 3^ MDM proteins are required to negatively regulate p53 activity in mice during the early and mid-stages of development, and embryonic lethality caused by MDM-deficiency is rescued by concomitant p53 deletion.^4, 5, 6, 7^ Mdm2 and MdmX also play critical roles in peri-natal and adult mice by controlling p53 regulation of cell differentiation, the DNA damage response, tissue homeostasis and aging.^8^

MDM2 and MDMX also regulate p53-mediated tumor suppression. *MDM* gene amplification and/or overexpression have been observed in a variety of human cancers, including leukemia and lymphoma, breast cancer, glioblastoma, soft tissue sarcoma, osteosarcoma and retinoblastoma.^9, 10, 11^ As most tumors with amplified copy numbers of *MDM* genes retain wild-type p53,^10, 12^ the increased level of MDM2 or MDMX proteins is thought to promote oncogenesis by inhibiting p53 activity in these cells. Experiments involving Mdm2 or MdmX overexpression in cells or mice also suggest that MDM oncoproteins may have p53-independent roles in regulating cell growth and tumorigenesis^13, 14, 15, 16, 17^ although the precise contributions of these p53-independent, proto-oncogenic effects in human cancers remains uncertain.

We have previously shown that mice co-deleted for both *MdmX* and *p53* succumb to spontaneous tumorigenesis faster than mice deleted solely for *p53*,^18^ suggesting p53-independent anti-oncogenic function for MdmX in mice. In contrast, deletion of *Mdm2* fails to alter the tumorigenic potential of mice lacking functional p53.^19^ Down-regulation or loss of functional MDMX protein has also been associated with more aggressive or advanced osteosarcomas, soft tissue sarcomas, thyroid and prostate carcinomas, and chronic myelogenous leukemia.^20, 21, 22, 23, 24^ In addition, an alternatively spliced MDMX variant is often found in high-grade glioblastomas, papillary thyroid carcinomas, soft tissue sarcomas and osteosarcomas.^20, 21, 24^ In both human tumors^20, 25^ and in mouse model with targeted *MdmX* internal deletion^26^ this altered splicing reduces the level of full-length (FL) MdmX transcript and generates a novel transcript encoding a severely truncated, unstable MdmX protein. The increase in short to FL transcript ratio in osteosarcomas correlates with reduced MDMX protein levels, faster metastatic progression and greatly reduced patient survival.^20^ Lower MDMX protein levels in many osteosarcoma or breast cancer cell lines and in soft tissue sarcomas correlate with compromised p53 function.^20^ Although it is likely that p53-mutant tumor cells have lost the selective pressure to maintain high levels of functional MDMX, it is unclear why loss of functional MDMX in these cells correlates with a more aggressive cancer.

We previously observed that p53-deficient mouse embryo fibroblasts (MEFs) and p53-deficient mouse tumor cells proliferate faster when *MdmX* is also deleted, and that MdmX/p53-double-null cells have increased incidence of multipolar mitosis and reduced cell ploidy compared with p53-null cells.^18^ These findings suggest a p53-independent role for MdmX in suppression of proliferation and in maintenance of genome stability in hyperploid mouse cells. In the present study, we use human tumor cells in mouse orthotopic transplantation and lung colonization assays to explore the relevance of these p53-independent effects of MdmX in tumorigenesis. We provide the evidence that MdmX suppresses tumor progression and metastases in these mouse models of human cancer. Furthermore, we find the inhibition of cell proliferation and maintenance of genome stability to be separable MdmX functions encoded by different MdmX protein domains. We demonstrate that the ability of MdmX Zn-finger domain to suppress multipolar mitosis and large-scale ploidy reduction in p53-mutant cells underlies the role of MdmX in tumor suppression. We discuss the implications of our findings on cancer treatment strategies and on current models of genome instability and cancer progression.

## Results

### MdmX slows cycling of p53-deficient cells

**Figure 1:**
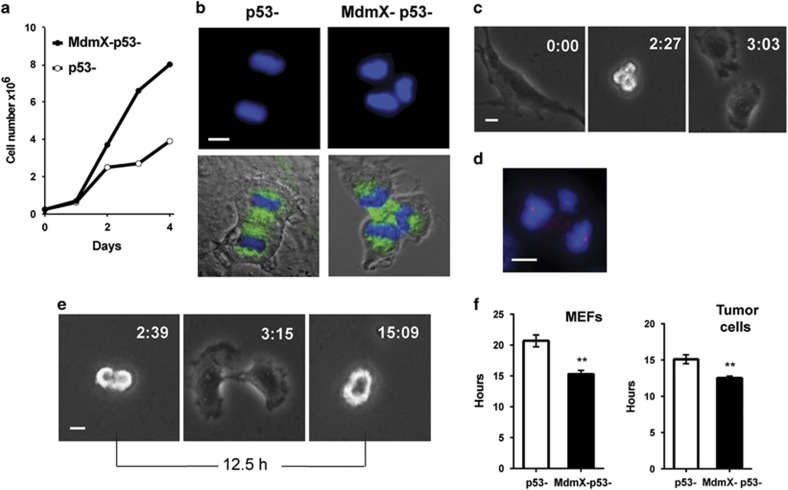
MdmX slows cycling of p53-deficient cells. (**a**) Proliferation of p53-null mouse tumor cells bearing or lacking MdmX. (**b**) Immunofluorescence staining of mouse tumor cells illustrating bipolar (left panels) and tripolar (right panels) anaphase. Cells were stained for alpha-tubulin (green) and for DNA (4′-6-diamidino-2-phenylindole (DAPI), blue). (**c**) Images from time lapse video microscopy of MdmX/p53-null tumor cells undergoing tripolar mitosis and generating two viable daughter cells. (**d**) Immunofluorescence staining for gamma-tubulin (red) and DNA (DAPI, blue) of MdmX/p53-null cell undergoing multipolar mitosis with uneven distribution of genetic material. (**e**) Images from time lapse video microscopy illustrating measurements of cell cycle length. (**f**) Quantification of cell cycle length in MEFs (left panel) and mouse tumor cells (right panel). Error bars represent mean±s.e.m. from 50 to 100 cell divisions scored for each cell line (**P<0.0001, unpaired *t*-test). Scale bars, 10 μm.

MdmX/p53 double-null MEFs and primary epithelial tumor cells from MdmX/p53 double-null mice proliferate faster than MEFs and tumor cells solely deficient for p53 (ref. 18 and Figure 1a). Multipolar mitosis (Figure 1b) are more common in populations of MdmX/p53-double-null than in p53-null cells (20% vs 10%, respectively, of all mitotic cells). Therefore, it is possible that the divisions that generate more than two daughter cells per division might contribute to the increased proliferation rate of MdmX/p53-null cells. We have previously demonstrated^27^ that polyploid cells undergoing multipolar mitosis can indeed generate more than two daughter cells but many of the resulting progeny dies during one or two subsequent divisions. Time-lapse video microscopy analyses now revealed that only 21% of all multipolar mitosis results in multipolar division and 71% of such progeny died or arrested during the 69 h of filming. A majority (79%) of multipolar mitosis produced only two viable daughter cells (Figure 1c) that underwent normal bipolar mitosis and continued to divide in bipolar fashion until the end of filming. Gamma-tubulin/4′-6-diamidino-2-phenylindole–staining of cells in late multipolar anaphase typically revealed an unequal distribution of genetic material illustrated in Figure 1d. Therefore, it is unlikely that multipolar mitosis and the generation of more than two daughter cells per division accounts for faster proliferation rate of MdmX/p53-null cells. We applied live imaging to determine the duration of cell cycle at the single-cell level by measuring the length of time from the onset of anaphase in mother cell to the onset of anaphase in daughter cells (Figure 1e). The results showed that the absence of MdmX in p53-deficient cells significantly shortens cell cycle length (Figure 1f). MEFs (left panel) or tumor cells (right panel) lacking both MdmX and p53 cycled 5 h and 2.5 h faster, respectively, than their p53-deficient counterparts retaining MdmX. Thus, it is a more rapid cell cycle progression and not a higher number of daughter cells per division that accounts for the faster proliferation rates of p53-deficient cells co-deleted for *MdmX*.

**Figure 2:**
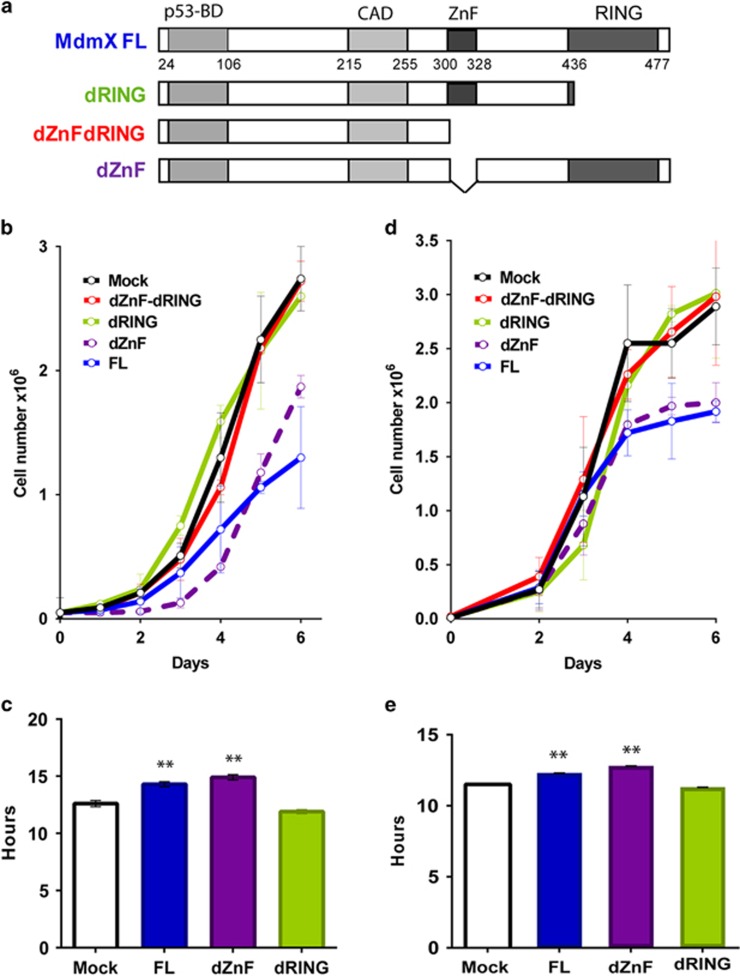
MdmX RING domain suppresses proliferation of p53-null cells. (**a**) Schematic representation of mouse MdmX protein domains and deletion mutants used in this study. (**b**) Proliferation of MdmX/p53-double knockout (DKO) mouse tumor cells transduced with MdmX FL or deletion mutants. Error bars represent mean±s.d. from two to three independent experiments. (**c**) Cell cycle length of MdmX-transduced DKO cells measured by time lapse video microscopy. Error bars represent mean±s.e.m. from 50 to 100 cell divisions scored for each cell line. (**d**) Proliferation of transduced MdmX/Mdm2/p53-triple knockout (TKO) tumor cells. Error bars represent mean±s.d. from three to five independent experiments. (**e**) Cell cycle length of MdmX-transduced TKO cells measured by time lapse microscopy. At least 100 cell divisions were scored for each genotype. *P*-values relative to Mock control: **P<0.001, unpaired *t*-test.

The primary sequence of MdmX contains four functional domains: an amino-terminal p53 binding (p53-BD) domain, a central acidic domain, a zinc-finger (ZnF) domain of unknown function and a carboxy-terminal RING domain critical for the interaction of MdmX with Mdm2. To characterize the ability of MdmX to suppress cell proliferation and multipolar mitosis, we utilized mouse MdmX expression constructs encoding either the FL, or mutant MdmX lacking carboxy-terminal RING domain (dRING), central zinc-finger domain (dZnF) or both (dZnF-dRING; Figure 2a). These constructs were co-transfected along with a puromycin drug selection marker into the epithelial tumor cells derived from MdmX/p53-double knockout (DKO) mice, and pools of puromycin-resistant stably transfected cells were recovered for further analyses.

Quantitative PCR analyses showed that the levels of MdmX messenger RNA (mRNA) in transfected cells (FL, 14.2±5.7, dRING, 11.0±5.8; dZnF, 13.3±3.6; dZnF-dRING, 35.2±4.5) are similar to the endogenous MdmX levels in p53 null mouse tumor cells (13.7±4.5) and in wild-type MEFs (11.07±4.6). Proliferation assays revealed that exogenous FL MdmX and dZnF mutant suppress proliferation of MdmX/p53-deficient cells (Figure 2b). In contrast, MdmX lacking either the RING domain or lacking both the ZnF and RING domain failed to alter the proliferation of the double-null cells. These results indicate that the RING domain of MdmX encodes an anti-proliferative function. The role of the MdmX RING domain in suppressing cell proliferation was confirmed at the single-cell level by video microscopy (Figure 2c). The presence of MdmX RING domain, but not the central ZnF domain, correlates with an ~2 h increase in cell cycle duration of p53-null cells.

Since MdmX interacts with Mdm2 and forms heterodimer through its RING domain, we explored whether the p53-independent effect of RING domain on cell proliferation requires Mdm2. Tumor cells derived from Mdm2/MdmX/p53-triple deficient (TKO) mice were transduced with FL or MdmX deletion constructs (relative MdmX mRNA levels: Mock, 1.0; FL, 6.6±3.6; dRING, 12.1±7.0; dZnF, 23.5±2.8; dZnF-dRING, 7.4±2.3). Cell proliferation assay (Figure 2d) reveal that MdmX with intact RING domain suppresses proliferation of TKO cells. Analysis by video microscopy confirmed that the presence of RING domain prolonged the cell cycling time from 11.5 h (Mock) to 12.3 h (FL) or 12.8 h (dZnF) in TKO cells (Figure 2e). These results demonstrate that the ability of MdmX to suppress proliferation of p53-deficient cells does not require functional Mdm2.

### Zn-Finger domain of MdmX maintains genome stability in mouse cells

**Figure 3:**
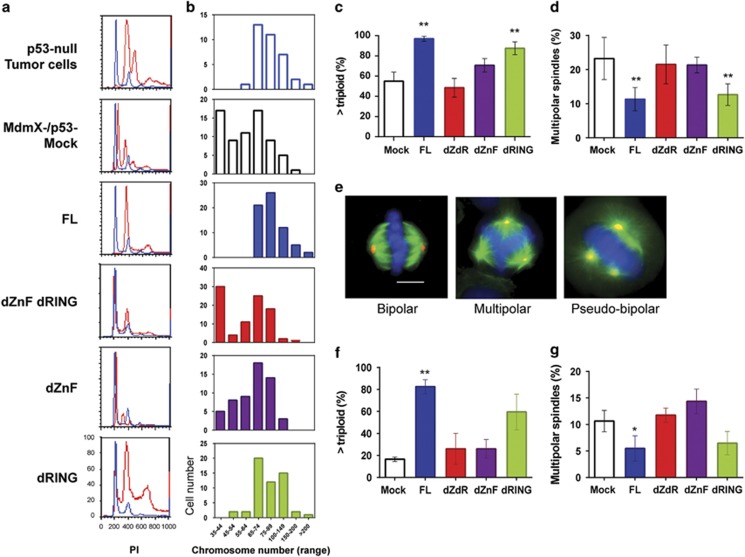
Zn-finger domain of MdmX suppresses genome instability in mouse tumor cells. (**a**) DNA content of MdmX/p53-double knockout (DKO) mouse tumor cells transduced with MdmX FL or deletion mutants determined by propidium iodide staining. DNA content of experimental cells (red histograms) was superimposed over the DNA content of parental non-transduced MdmX/p53-null cells (blue histograms). (**b**) Metaphase spread analysis for chromosome number per cell (in indicated ranges) in MdmX-transduced DKO cells. The results are from representative experiment with at least 50 metaphase cells scored per cell line. (**c**) Summary of chromosome analyses showing cell fraction with larger than triploid genome in population of transduced DKO cells. Error bars represent mean±s.d. from two to four independent experiments with more than 50 metaphase cells scored per cell line per experiment. (**d**) Frequency of multipolar spindles as a percent of total mitotic events in the populations of MdmX-transduced DKO cells. More than 200 mitotic spindles were scored per experiment. Error bars represent mean±s.d. from three independent experiments. (**e**) Representative imunofluorescence images of DKO cells illustrating mitotic spindle organization. Cells were stained for alpha-tubulin (green), gamma-tubulin (red) and DNA (4′-6-diamidino-2-phenylindole, blue). Scale bar, 10 μm. (**f**) Chromosome analyses showing cell fraction with larger than triploid genome in population of MdmX-transduced MdmX/Mdm2/p53-triple knockout (TKO) mouse tumor cells. Error bars represent mean±s.d. from two independent experiments with at least 50 metaphases scored per cell line per experiment. (**g**) Frequency of multipolar spindles expressed as a percent of total mitotic events in the populations of transduced TKO cells. Error bars represent mean±s.d. from two to three independent experiments. *P-*values relative to Mock control: **P<0.005; *P from 0.005 to 0.05, unpaired *t*-test.

We have reported that MdmX inhibits multipolar mitosis and chromosome loss in hyperploid p53-deficient cells.^18^ To identify the domain(s) in MdmX protein responsible for promoting genome stability, we analyzed the DNA content and chromosome number of mouse DKO tumor cells transduced with MdmX expression constructs. Propidium iodide staining and metaphase spread analysis indicate that the expression of FL MdmX in DKO cells increased DNA content and chromosome numbers to the levels seen in tumor cells from p53-null mice retaining endogenous MdmX (Figures 3a and b). This MdmX-mediated increase in ploidy of DKO cells does not require the RING domain (see Figure 3a and b, FL and dRING panels), but it does require ZnF domain of MdmX (see Figure 3a and b, dZnF-dRING and dZnF panels). Metaphase spread analyses summarized in Figure 3c show that nearly the entire population of DKO cells with intact ZnF domain contained more than 60 chromosomes per cell, while the deletion of ZnF domain abolished this increase in chromosome number.

We have proposed that genome instability in hyperploid p53-deficient cells lacking MdmX arises from aberrant chromosome segregation and loss during multipolar mitosis, an event that occurs much more frequently in p53-null cells that also lack MdmX.^18^ We therefore expect that the suppression of chromosome loss by the Zn-finger domain may be linked to the suppression of multipolar mitosis. The immunofluorescence analyses of spindle formation in DKO cells (Figure 3d) showed that the expression of MdmX with intact ZnF domain reduced the frequency of multipolar spindles by 50%. In contrast, expression of MdmX lacking ZnF domain failed to decrease the incidence of multipolar mitotic events. In Figure 3e, examples of normal bipolar mitosis (left panel), multipolar mitosis represented by tri-polar event (middle panel) and pseudo-bipolar mitosis with centrosomes clustered around two opposite poles (right panel) are shown. These results reveal that the ability of MdmX to prevent chromosome loss and to suppress multipolar mitosis both require the MdmX Zn-finger domain. To confirm that MdmX function in genome stability does not require Mdm2, the experiments were repeated using the TKO tumor cells transduced with the MdmX expression constructs. The results of chromosome analyses (Figure 3f) and mitotic spindle analyses (Figure 3g) confirmed that Mdm2 is not required for MdmX to regulate genome stability.

### The Zn-finger domain of MdmX maintains genome stability in p53-deficient human cells

**Figure 4:**
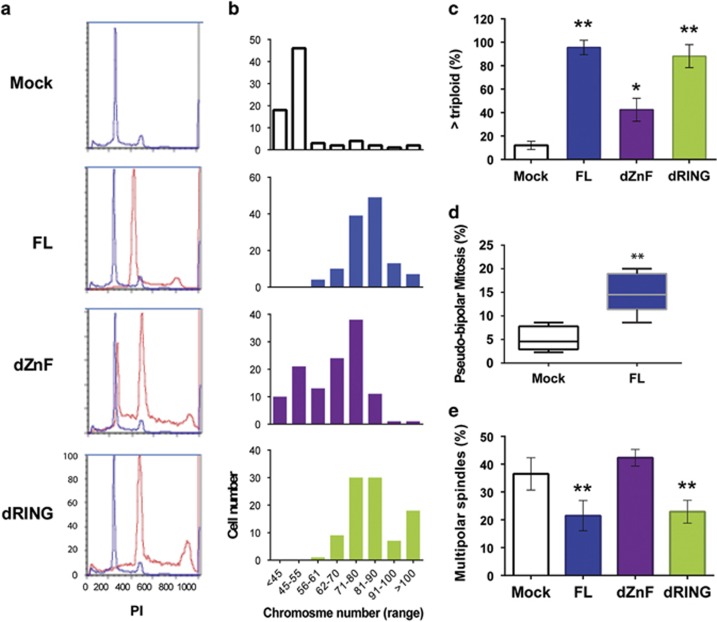
Zn-finger domain of MdmX suppresses genome instability in p53-deficient human cells. (**a**) DNA content determined by propidium iodide staining of human breast tumor MB157 cells transduced with MdmX FL or deletion mutants. DNA content of experimental cells (red histograms) was superimposed over the DNA content of Mock control cells (blue histograms). (**b**) Metaphase spread analysis for chromosome number per cell (in indicated ranges) in transduced MB157 human breast tumor cells. At least 50 metaphases were scored per cell line. (**c**) Summary of chromosome analyses showing cell fraction with larger than triploid genome in population of transduced MB157 cells. Error bars represent mean±s.d. from three to four experiments with at least 50 metaphase cells scored per experiment. (**d**) Frequency of pseudo-bipolar mitosis expressed as a percent of total mitotic events in population of MdmX-transduced MB157 cells. Spindles with amplified centrosomes clustered around the opposite pools (see Figure 3d, right panel) were scored as pseudo-bipolar mitosis. Results from five experiments with more than 100 mitotic events per experiment are shown in Box-whiskers plots. (**e**) Frequency of multipolar spindles in the populations of transduced MB157 cells. More than 200 mitotic spindles were scored per experiment. Error bars represent mean±s.d. from average of five experiments for each cell line. *P-*values relative to Mock control: **P<0.005; *P from 0.005 to 0.05, unpaired *t*-test.

To determine if MdmX has a p53-independent role in regulating genome stability in human cells, we utilized MB157 breast tumor cells that bear a truncating deletion in *TP53* gene^28^ transduced with MdmX expression constructs. Propidium iodide staining and metaphase spread analyses revealed that the presence of either FL or mutant MdmX lacking the RING domain increased DNA content (Figure 4a) and chromosome number (Figure 4b) relative to the Mock control or to cells with MdmX lacking Zn-finger domain. Evaluating the number of cells with genome exceeding a triploid chromosome content substantiated a role for MdmX ZnF domain in promoting genome stability in p53-null human breast cancer cells (Figure 4c).

To determine if the MdmX ZnF domain regulates spindle polarity in human tumor cells, we performed immunofluorescence staining and analyzed spindle formation. Population of MB157 cells displayed high rate of multipolar mitosis and supernumerary centrosomes, facilitating measurements of pseudo-bipolar mitotic events. Formation of pseudo-bipolar spindles increased three-fold when FL MdmX was expressed (Figure 4d) and this increase correlated with the reduced number of multipolar mitotic evens (Figure 4e). As in mouse cells, in MB157 human cells MdmX-mediated suppression of chromosome loss correlates with the suppression of multipolar mitosis and they both require intact ZnF domain.

### MdmX Zn-finger domain suppresses tumor growth

**Figure 5:**
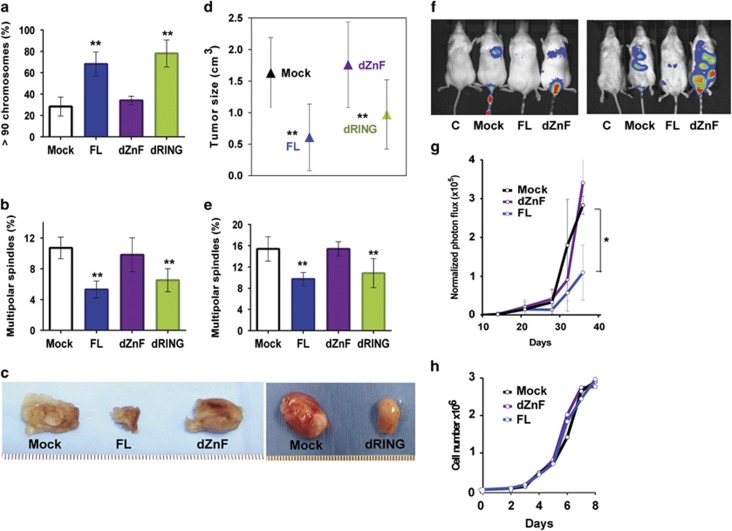
The Zn-finger domain of MdmX suppresses tumor growth and metastatic potential of human tumor cells. (**a**) Chromosome analyses showing percent of cells with >90 chromosomes per cell in populations of human MB231 breast tumor cells transduced with MdmX FL or deletion mutants. Error bars represent mean±s.d. from four to six mitotic spreads with at least 30 metaphase cells scored per cell line per spread. (**b**) Frequency of multipolar spindles in populations of transduced MB231 cells. More than 200 mitotic events were scored per cell line per experiment. Error bars represent mean±s.d. from four experiments. (**c**) Tumors formed after orthotopic transplantation of MB231 cells into the mammary fat pad of nude mice. Tumors were harvested 16 days after transplantation. (**d**) Tumorigenic potential of transduced MB231 cells transplanted into the fat pad of nude mice. Tumor size was calculated from equation: cm^3^=½ (W^2^ × L). The error bars represent mean±s.d. of tumor size from 3 to 5 animals per each cell line. (**e**) Frequency of multipolar spindles in populations of cells cultured from tumors harvested after transplantation of transduced MB231 cells into the mammary fat pad of nude mice. More than 500 mitotic events were scored in population of cells deriving from each tumor. Error bars represent mean±s.d. from three to five tumors. (**f**) Representative bioluminescence images of lung colonization in mice. MdmX-transduced MB231 cells infected with retroviral M-cherry-Luciferase reporter were injected into the tail vein of nude mice. Lung colonization was assayed by bioluminescence imaging following intraperitoneal injection of Luciferin. From left to right: control for auto-luminescence (cells without retrovirus); Mock (cells without MdmX); cells with FL MdmX; cells with MdmX-dZnF mutant. (**g**) Metastatic potential of transduced MB231 cells expressing Luciferase reporter, injected into the tail vein of nude mice (2.5 × 10^5^ cells per animal, five animals per group). Luciferase activity was monitored over time using IVIS 100 imager. Intensity of photon flux within the region of interest from combined dorsal and ventral images of animals within the group was normalized to the intensity of photon flux on day 1. (**h**) Proliferation of MdmX-transduced MB321 cells used in experiments presented in **a**–**g**. *P*-values relative to Mock control: **P<0.005; *P value from 0.05 to 0.005, unpaired *t*-test.

To determine if ZnF-dependent abilities of MdmX to suppress multipolar mitosis and chromosomal loss can impact tumor progression, we employed MB231 cells, a p53-mutant human breast cancer cell line frequently used in orthotopic tumor transplantation studies transduced with MdmX constructs. Exogenous mouse MdmX mRNA levels in transduced cells determined by quantitative PCR analyses normalized to Mock are: FL, 8.2±1.8; dRING, 16.4±4.0; and dZnF, 15.4±7.2. These values are in the same range as the endogenous human MdmX level (15.2±5.3) measured in the same cells. We first confirmed that MdmX also inhibits chromosome loss (Figure 5a) and multipolar spindle formation (Figure 5b) in these cells only when Zn-finger domain is intact.

For *in vivo* experiment, cells were transplanted into the mammary fat pad of immune-compromised NSG (NOD-scid IL2ry null) mice and tumors were harvested and measured 16 days later. Tumors developed in all animals, but were much smaller in mice implanted with MdmX-FL and MdmX-dRING-containing cells than in mice with mock-control or MdmX-dZnF cells (Figure 5c). The analysis of tumor size confirmed the suppressive effect of the ZnF domain on tumor progression (Figure 5d). Following tumor dissection, cells were isolated and cultured for the spindle analyses. Immunofluorescence staining confirmed that the suppression of tumor growth with cells expressing intact Zn-finger domain correlates with a reduction in number of multipolar spindles before and after transplantation (Figure 5e).

### MdmX suppresses metastatic potential of human cancer cells

To investigate the effect of MdmX on metastatic potential of MB231 cells, we utilized whole-body *in vivo* bioluminescence imaging and a dual reporter gene system. Mock-control cells or cells transduced with MdmX-FL or MdmX-dZnF constructs were infected with retrovirus encoding a FUW-M-cherry-Luciferase reporter gene and sorted for M-cherry positive expression by FACS. Robust expression of M-cherry was confirmed by fluorescence microscopy. Cells were injected into the tail-vein of NSG mice, and lung colonization by MB231 cells was monitored over time by whole-body imaging for luciferase activity. Representative images of bioluminescence signal at various time points are shown in Figure 5F. The intensity of photon flux in the lungs of mice at different time intervals was normalized to the intensity on day 1 (Figure 5g). The signal intensity increased over time in all experimental animals injected with the reporter-bearing cells. However, the colonization and growth of MB231 cells in the lungs of mice were much slower when the injected cells expressed MdmX with intact ZnF domain. Thus, the ability of MdmX to inhibit both tumor progression and metastatic lung colonization by human tumor cells in mice correlates with the presence of the MdmX-central Zn finger domain. The deletion of Zn-finger domain does not affect proliferation of MdmX-transduced MB231 cells *in vitro* (Figure 5h) suggesting that the proliferation rate itself does not underlay the differences in tumorigenic potential observed *in vivo*.

## Discussion

*MDMX* is frequently amplified and overexpressed in human cancers, and experiments utilizing human cells and genetically altered mice have established that the oncogenic potential of MdmX is largely due to its ability to inhibit the p53 tumor suppressor. However, our lab has previously noted the existence of p53-independent roles for MdmX in suppressing cell proliferation and in maintaining genome stability^18, 29^ suggesting that MdmX may have both oncogenic activity (via p53 inhibition) and anti-oncogenic capabilities (in tumors mutated for p53). Here, we establish that the carboxy-terminal RING domain of MdmX prolongs the duration of cell cycle in p53-deficient cells, thereby suppressing cell proliferation. We also determine that MdmX-mediated suppression of multipolar mitosis correlates with increased genome stability in hyperploid mouse and human tumor cells. This p53-independent function of MdmX maps to the central Zn-finger domain of the MdmX protein that matches the consensus sequence for the Ran binding protein 2 (RanBP2) type zinc fingers (ScanProsite results, release 20.119 of 12 October 2015, score 9.077). The Zn-fingers domain in RanBP2 nucleoporin facilitates its binding to the small GTPase Ran protein^30^ which is associated with nuclear envelop assembly and mitotic spindle morphogenesis.^31, 32^ It can be speculated that MdmX demonstrates its centrosome clustering function as one of the downstream targets for Run in spindle formation. As such, MdmX might counteract the centrosome clustering inhibitory activity of previously identified Ran target, nuclear mitotic apparatus protein (Nu-MA).^33, 34^ Both MdmX and Mdm2 proteins have conserved RanBP2 zinc finger-like consensus sequences. However, there is only 43% identity in amino acid sequence in Zn-finger domain between the two proteins that may account for the difference in their role in p53-independent tumorigenesis.

Increased cell proliferation and chromosomal instability are hallmarks of tumorigenesis. Since we determined that the suppression of proliferation and genome instability by MdmX are independently regulated, we were able to examine relative contributions of these activities to MdmX-mediated suppression of tumorigenesis. Utilizing standard orthotopic transplantation assays to measure tumor growth, and lung colonization by *in vivo* imaging to measure metastatic activity, we determined that MdmX-mediated genome stabilization underlies the ability of MdmX to reduce tumorigenic potential of cells mutated for p53. Although we cannot formally exclude a role for MdmX-mediated inhibition of cell proliferation in tumor progression, it is noteworthy that exogenous MdmX fails to alter proliferative capacity of MB231 cells *in vitro*, yet it clearly suppresses tumor formation and metastasis *in vivo*. It suggests that the tumorigenic potential of hyperploid, p53-mutant cells is not driven by a proliferative advantage but rather by the genomic instability imparted by loss of MdmX.

Computational quantification of somatic DNA alterations in human cancers reveals that tetraploidy generated by whole-genome doubling events occurs frequently during tumorigenesis.^35^ Furthermore, polyploidy associated with increased genetic instability promotes aneuploidy and accelerated tumorigenesis in human cancers.^36, 37^ Pan-cancer analyses of somatic copy number alteration revealed that the whole-genome doubling occurred in 37% of cancers with average estimated ploidy of 3.31, suggesting the large-scale genome loss being associated with whole-genome doubling.^38^ However, experiments with long-term culturing of tetraploid cells deriving from diploid cancer progenitors^39^ revealed that, on per chromosome basis, tetraploidization by itself does not trigger additional instability. Dewhurst *et al.*^39^ suggest that a tetraploid genome increases cell tolerance to segregation errors and aneuploidy. In agreement with this, our data provide direct evidence that it is not the presence of a large genome *per se* but rather the ability of these hyperploids to tolerate a greater range of genetic imbalances leading to the evolution of aggressive growth characteristics. We propose that MdmX exercises its tumor suppressive activity in hyperploid cells with compromised p53 by preventing multipolar mitosis and stabilizing the large genome of these cells, thus preventing chromosome loss. Loss of MdmX may trigger large-scale ploidy reductions (only tolerated in polyploid cells), reducing the ploidy to near-diploid levels until a new cycle of polyploidization occurs within the cell. Such a scenario of non-synchronized ‘genomic breathing' in populations of tumor cells might easily contribute to tumor heterogeneity, and is concordant with the model of parallel evolution of different subclones within the same tumor.^40, 41^ Interestingly, overexpression of MdmX was recently shown to impair DNA damage response and promote chromosome and chromatid breaks in mouse embryo fibroblasts *in vitro*.^42^ In contrast to those aberrations that occur at the level of individual chromosome(s), the genomic instability imparted by the loss of MdmX that we refer to represents the large-scale genome loss restricted to the polyploid cells that can lead to aneuploidy and tumorigenesis. These two quantitatively and mechanistically distinct MdmX-mediated processes are not mutually exclusive and are likely dependent on cellular context.

Our findings also suggest that genome stabilization by MdmX is dependent upon the clustering of supernumerary centrosomes and the promotion of pseudo-bipolar mitosis. Although centrosome amplification and clustering is not limited to cancer cells,^43^ it is believed that the centrosome clustering represents major route for hyperploid cancer cells with amplified centrosomes to escape multipolar mitosis and survive. Not surprisingly, identification and the inhibition of proteins involved in centrosome clustering has become an attractive anticancer strategy.^44, 45^ However, our experiments reveal that the majority of multipolar mitosis generates viable daughter cells with increased levels of aneuploidy and increased tumorigenic potential. Thus, our findings offer a caveat to proposed cancer treatment strategies that aim to promote multipolar mitosis. Furthermore, our data indicate that the loss of MdmX in p53-mutant cells promotes genome instability and cancer progression. As suggested by recent studies demonstrating the failure of anti-MdmX strategy in tumors expressing hypomorphic p53 mutant,^46^ our data indicate that cancer treatments centered on MdmX inhibition should be employed only in cases where tumor retains functional p53.

## Materials and methods

### Cells and cell culture

MEFs were isolated from 13.5-day-old embryos deriving from crosses between *MdmX+/−, p53+/−* mice and *MdmX+/−, p53−/−* mice. Mouse tumor cells were isolated from thymic tumors of *MdmX−/−, p53−/−* mice or *p53−/−* mice and genotyped by PCR. The MdmX mouse model used in this study previously obtained by gene-trapping^47^ does not generate a detectable MdmX protein.^6^ Mouse cells were grown at 37 °C with 5% CO_2_ in Dulbecco's modified Eagle medium (DMEM) supplemented with 10% fetal bovine serum (FBS), penicillin and streptomycin.

Human tumor cell lines MDA-MB157 (obtained from ATCC) and MDA-MB231 (gift from Dr. Jane Lian, UMASS Medical School) were established from metastatic pleural effusion of human breast carcinomas.^48^ MB231 cells were grown at 37 °C with 5% CO_2_ in DMEM with penicillin, streptomycin and 10% serum. For MB157 cells media was supplemented with 15% serum. Cell line MB231 has missense mutation in exon 8 resulting in mutant p53 protein^49^ and it is tumorigenic in nude mice.^50^ MB157 cells are p53-deficient due to the deletion in *TP53* exon 4 resulting in the absence of p53 protein.^28^ MB157 cells have hardly detectable levels of MDMX protein.^51^ All cells were tested and were free of mycoplasma.

### Plasmids

For the transfection experiments, murine *MdmX* complementary DNA (cDNA) was placed under the transcriptional control of a conjugate promoter bearing a cytomegaloviral enhancer and chicken-B actin promoter sequences (pCAGGS). Construction of MdmX expression plasmids with MdmX FL and MdmX deletion mutants MdmX-dRING (amino acids 1–444), and MdmX-dZnF-dRING (amino acids 1–300) was described previously.^52^ Those plasmids were generous gift from Steven J. Berberich (Wright State University). Deletion mutant MdmX-dZnF (d304-323) was generated from the wild-type *MdmX* cDNA by site-directed mutagenesis and confirmed by DNA sequencing (GenScript, Piscataway NJ, USA).

### Culturing cells from tumors

Following tumor dissection, tumors were minced in high glucose DMEM supplemented with antibiotics and collagenase (2mg/ml), incubated at 37°C for 3 h with shaking, washed with PBS containing 5% FBS, resuspended in DMEM with antibiotics and 2% FBS and plated on collagen-coated plates. Cells were maintained in 2% FBS DMEM/F12 with antibiotics. Fibroblasts were removed by differential trypsinization. Once cells reached growth crises, cells were fed with low glucose 10% FBS DMEM.

### Transfection of mouse and human cells

Cells were grown in DMEM with no antibiotics until 70–80% confluent. Linearized MdmX-expression constructs were co-transfected with PGK promoter-puromycin drug selection marker using Lipofectamine 2000 (Invitrogen, Carlsbad, CA, USA) for transfection of mouse and MDA-MB231 human cells or XtremeGene-9 (Roche, Mannheim, Germany) for MDA-MB157 human cells. Cells were split 24 h after transfection and selection for puromycin resistance started 24 h later. Pools of puromycin-resistant stably transfected clones were recovered for further analyses.

### Quantitative real-time PCR

Total mRNA was isolated using RNeasy kit (Qiagen, Hilden, Germany). For the first-strand cDNA synthesis SuperScript III First-Strand Synthesis System (Invitrogen) was used according to manufacturer's instructions. Quantitative PCR reaction was performed with SYBR Select Master Mix (Invitrogen) using the ABI-9300 PCR machine. The MdmX mRNA data were normalized to actin and the level in Mock control was assigned a value 1. Primers used for quantitative PCR are as follows:

Mouse MdmX forward, 5′-TCTCGCACAGGATCACACTATGGA-3′;

Mouse MdmX reverse, 5′-TCATCTGCTCTGGAGTCTCTGCAT-3′;

Mouse Actin forward, 5′-TCCTGTGGCATCCATGAAACT-3′

Mouse Actin reverse, 5′-GAAGCACTTGCGGTGCACGAT-3′

Human MdmX forward, 5′-GCAAGAAATTTAACTCTCCAAGCAA-3′

Human mdmX reverse, 5′-CTTTGAACAATCTGAATACCAATCCTT-3′

Human Actin forward, 5′-GGACTTCGAGCAAGAGATGG-3′;

Human Actin reverse, 5′-AGCACTGTGTTGGCGTACAG-3′.

### Time-lapse video microscopy

Cells were plated on coverslips 24 h before filming in DMEM media with 10% serum, penicillin, streptomycin and 12.5 mM Hepes. Coverslips with attached cells were assembled into the sealed chambers^53^ and incubated at 37 °C. Individual cells were observed under the Zeiss Universal or Leica DMEXE microscope equipped with phase-contrast optics using × 10 objectives. Images were recorded with Orca ER, Orca100 (Hamamatsu, Bridgewater, NJ, USA) Retiga EX and/or Retiga EXi cameras (Qimaging, Surrey, BC, Canada), acquired every 3 minutes with C-imaging software and exported as a QuickTime movies (100% of CinePak compression mode). The duration of cell cycle was defined as the time from anaphase onset in mother cell manifested as the chromosome disjunction and/or sudden start of cell elongation till the anaphase onset in daughter cells.

### Immunofluorescent microscopy

Cells were grown on glass coverslips, fixed in methanol and stained for microtubules with mouse monoclonal anti-α-tubulin (Sigma-Aldrich, T5168) and for centrosomes with rabbit polyclonal anti-γ-tubulin (Sigma-Aldrich, T5192) primary antibodies. Alexa488 and Alexa594 (Molecular Probes, A11001 and A11012) were used as secondary antibodies. DNA was stained with 4′-6-diamidino-2-phenylindole. Immunofluorescence images were recorded using a CCD camera attached to an epifluorescence Zeiss Axioplan 2 microscope (Zeiss, Thornwood, NY, USA). Single images were acquired using Metamorph Imaging Software (Universal Imaging, Downingtown, PA, USA).

### Metaphase spreads and chromosome counting

Actively proliferating cells were incubated with Colcemid (Gibco; 0.02 μg/ml media) for 90 min, harvested and incubated in hypotonic solution (0.075 M KCl) for 15 min at 37 °C and fixed with methanol-acetic acid. Drops of cell suspension were placed on microscope slides, dried, 4′-6-diamidino-2-phenylindole stained and analyzed by fluorescent microscopy.

### Orthotopic transplantation studies

MDA-MB231 cells stably transduced with FL or MdmX deletion mutants were resuspended in PBS with 30% Matrigel (BD Biosciences, San Jose, CA, USA). Cells (4 × 10^6^) in 200 μl were injected into the mammary fat pad of 4 weeks old NSG mice, five animals per group. Tumor growth was monitored externally and animals were killed 16 days after injection. Tumors were harvested and tumor volume was calculated: total volume=½ (length x width^2^).^54^ All animal work in this manuscript was approved by the Institutional Animal Care and Use Committee of UMMS.

### Viral transduction of tumor cells

MDA-MB231 cells stable transduced with FL or MdmX deletion mutants in DMEM media without antibiotic with Polybrene (Sigma-Aldrich, St Louis, MO, USA; 8 μg/ml), 60–70% confluent, were infected with FUW based retroviral vector encoding dual M-cherry-Luciferase reporter (gift from Hong Zhang, UMMS). Cells were incubated at 37 °C for 48 h and the expression of M-cherry was confirmed by fluorescent microscopy. Cells were propagated, harvested with Accumax (Innovative Cell Technologies) and resuspended in sorting buffer (PBS with 1mM EDTA; 25 mM HEPES; 1% FBS and antibiotics) at 7 × 10^6^ cells/ml. Cells were sorted for positive M-cherry expression using FACSAria II cell sorter in Core Flow Cytometry facility at UMMS and propagated under standard conditions.

### *In vivo* bioluminescence imaging

For tail vein injection, cells were resuspended in PBS at 1 × 10^6^ cells/ml. Using a 26G needle, 200 μl of cells were injected into the tail vein of 8-week-old NSG mice, five animals per group. Immediately following injection, animals were imaged for the luciferase activity at time zero. D-Luciferin (Gold Biotechnology, St Louis, MO, USA; LUCK-500) was dissolved in DPBS (15 mg/ml), filter-sterilized and injected intraperitoneally (150 mg/kg body weight). Animals were anesthetized with isoflurane for 5 min and imaged from both dorsal and ventral position by Xenogen IVIS 100 Imaging System, Caliper Life Sciences in Optical Imaging Core Facility at UMMS. Mice were monitored for luciferase activity every other day for the first week and then weekly for the rest of the experiment. Bioluminescence images of upper dorsal region corresponding to the lung position were quantified using Living Image 2.60.1 software. The values for photon flux were normalized to those obtained at day 1.

### Statistical analysis

The two-sample two-sided unpaired *t*-test was used to compare the mean between two experimental conditions with the significance level 0.05 and confidence interval 95%. Number of samples and *P*-values for specific group of experiments are provided in figure legends. The normality of data were tested by the stem and leaf plot or by Kolmogorov–Smirnov test (for time-lapse video microscopy data). For animal studies sample size was estimated using Mead's resource equation with the degrees of freedom between 10 and 20. No blinding or randomization methods were used.
